# Arbuscular mycorrhizal fungal community abundance, functions, and symbiotic interactions revealed by root metatranscriptomes

**DOI:** 10.1002/imo2.70087

**Published:** 2026-02-21

**Authors:** Peilin Chen, John W. Taylor, Cheng Gao

**Affiliations:** ^1^ State Key Laboratory of Microbial Diversity and Innovative Utilization, Institute of Microbiology Chinese Academy of Sciences Beijing China; ^2^ College of Life Sciences University of Chinese Academy of Sciences Beijing China; ^3^ Department of Plant and Microbial Biology University of California Berkeley California USA

**Keywords:** arbuscular mycorrhizal fungal community, cytoskeleton, interaction, metatranscriptome, sexual reproduction

## Abstract

Arbuscular mycorrhizal (AM) fungal communities are often profiled by metabarcoding, but this approach can bias abundance estimates and provide limited insight into community function and interactions with the host. Here, using weekly, field‐based multi‐omics, we find metatranscriptomes and metagenomes increase estimates of AM fungal relative abundance 6−15‐fold over rDNA metabarcoding and that root AM fungal relative abundance correlates strongly with sorghum forage yield (*R* > 0.9) and moderately with grain yield/quality. AM fungal relative abundance is impacted directly by sorghum development and indirectly by drought via suppressed sorghum fatty acid gene expression. Gene co‐expression network revealed coordinated plant and AM fungal functions in structural reorganization, resource exchange, and signal communication between the symbiotic partners. We found that early‐season co‐transcriptions of AM fungal and host genes feature cytoskeletal genes essential for arbuscule formation, the fungal‐plant organs of nutrient and mineral exchange. Late‐season plants downregulate cytoskeletal genes, but AM fungi continue their upregulation, which is associated with fungal sporulation and putative mating. Our study identifies previously ignored massive underestimation of AM fungal relative abundance, highlights the predictive role of AM fungal relative abundance for crop productivity, and uncovers symbiotic life history dynamics underpinned by coupled and decoupled co‐expression of plant and AM fungal genes.

## Highlights

- Paradigm shift: PCR‐free methods reveal 6–15‐fold higher arbuscular mycorrhizal (AM) fungal abundance than metabarcoding, exposing systematic underestimation across decades of research.
- Predictive power: AM fungal abundance serves as a community‐level trait that predicts crop yield under drought conditions.
- Symbiotic dynamics: Symbiotic life history from arbuscule formation to sporulation is governed by coupled and decoupled co‐expression of plant and AM fungal genes.

## Graphical abstract

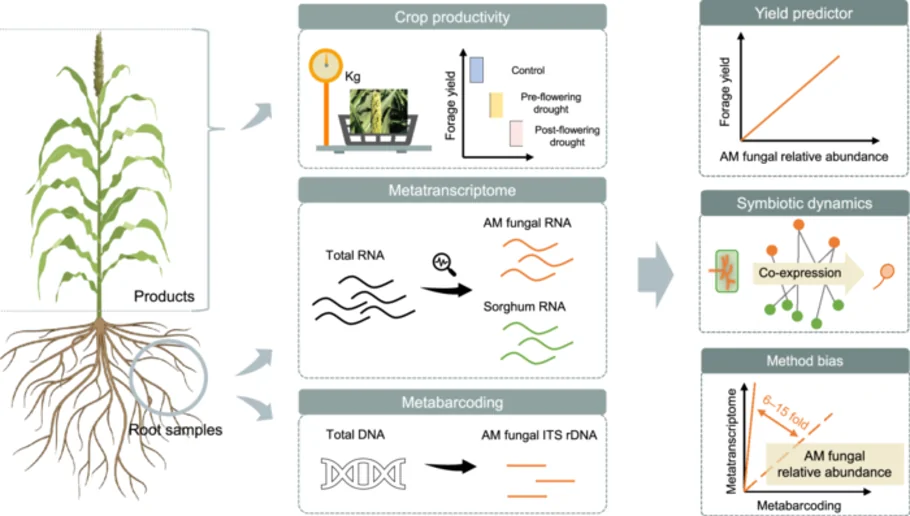

## 1 INTRODUCTION

Arbuscular mycorrhizas (AM) are among the most important, most ancient, and most prevalent mutualistic symbioses on Earth [1]. Preserved as fossils from ~400 million years ago, AM fungi now form symbioses with ~70–80% of terrestrial plant species, where they acquire and transport soil phosphorus to plants in exchange for 4% to 20% of the plant's photosynthate [1, 2, 3, 4, 5, 6]. What makes this complex system difficult to disentangle is the uncultivability, when separated from their host plant, of most AM fungi [7, 8]. Recently, the exploration of AM fungal function has added PCR‐free metatranscriptomes to a foundation based on pioneering greenhouse studies of AM fungal transcripts recovered from plant root transcriptomes (Table S1). As illuminating as these studies have been, most of them have used a single AM fungus and could not capture the complexity of natural systems that feature successional community dynamics of multiple AM fungi over plant development [9, 10, 11, 12, 13].

This knowledge gap extends to methodological discrepancies in detecting AM fungi in field settings. Although internal transcribed spacer (ITS) metabarcoding is widely used to profile fungal communities, it consistently reports surprisingly low AM fungal relative abundance [14, 15], which is unexpected given their established ecological significance [16, 17, 18]. Intriguingly, PCR‐free metatranscriptome data from mosses suggested higher Glomeromycota representation than ITS metabarcoding [19], though the symbiotic status of mosses remains debated [20]. Key uncertainties remain regarding AM fungal relative abundance detected by ITS metabarcoding in true AM hosts (>70% of vascular plant species, including most crops [21]). First, compared to PCR‐free approaches, it is unclear whether and to what extent ITS metabarcoding underestimates AM fungal relative abundance. Second, if ITS metabarcoding does underestimate AM fungal relative abundance compared to PCR‐free approaches, what drives this discrepancy? Third, the ecological relevance of AM fungal relative abundance itself is unclear; for example, does it correlate with plant productivity? We therefore hypothesize (H_1_) that ITS metabarcoding, as compared to PCR‐free approaches, underestimates AM fungal relative abundance.

The nexus for exchange of plant nutrients for fungal minerals, the arbuscule, is the root organ in which fungal hyphae penetrate root cortical cells without breaching plasma membranes. Arbuscule development requires coordinated structural remodeling of both symbionts, with cytoskeletal reorganization playing pivotal roles in this dynamic process [22, 23, 24, 25, 26, 27]. Recently, fostered by the characterization of metatranscriptomes of both AM fungi and their host plants (dual RNA‐seq), positive associations between the expressions of plant and AM fungal cytoskeleton genes have been reported in greenhouse studies using a single AM fungal genotype paired with a single cultivar of cassava or maize [28, 29]. Whether this coordination scales to complex field communities during crop development is unknown. Recognition of the importance of the arbuscule led us to propose the second hypothesis, H_2_, that transcriptional coordination of cytoskeleton‐related genes in both sorghum and AM fungal partners occurs throughout development in agricultural fields.

In this study, we integrated metatranscriptome, metagenome, and ITS2 metabarcoding datasets from sorghum roots, rhizosphere, and bulk soil collected weekly over a 17‐week field growing season (Figure S1). This multi‐omics framework rigorously tests our hypotheses, H_1_ that ITS metabarcoding, as compared to PCR‐free approaches, underestimates AM fungal relative abundance; and H_2_ that transcriptional coordination of cytoskeleton‐related genes in both sorghum and AM fungal partners occurs throughout development in agricultural fields.

## 2 RESULTS AND DISCUSSION

### Testing H_1_: PCR‐free methods reveal higher AM fungal relative abundance than metabarcoding

Using Kaiju, we quantified fungal and AM fungal reads in metagenome and metatranscriptome datasets by translating nucleic acid sequences into proteins and comparing them against protein databases. Based on the latest AM fungal phylogeny [30], we re‐analyzed the ITS2 metabarcoding dataset to update the taxonomic identification of 65 AM fungal operational taxonomic units (OTUs) (Figures S2, S3, and Table S2). Subsequently, we assessed the consistency of estimates of AM fungal relative abundance (i.e., the proportion of AM fungi within the total fungal community) across these three datasets (metatranscriptome, metagenome, and metabarcoding).

In roots, AM fungi constituted a median of 2.5% (range: 0.1–82.8%) of total fungal reads in the metatranscriptome dataset versus 0.7% (range: 0.04–13.9%) in the ITS metabarcoding dataset (Figure 1A and Figure S4). Switching to rhizosphere and soil samples, AM fungi represented 25.6% (range: 2.5–65.7%) of total fungal reads in the metatranscriptome dataset and 33.6% (range: 3.0–73.6%) in the metagenome dataset, a ratio that is essentially 1:1 (Figure 1B–D). As with roots, the estimate of relative AM fungal abundance based on ITS metabarcoding in rhizosphere and soil was much lower, 1.5% (range: 0.1–8.4%; Figure 1B–D).

**Figure 1:**
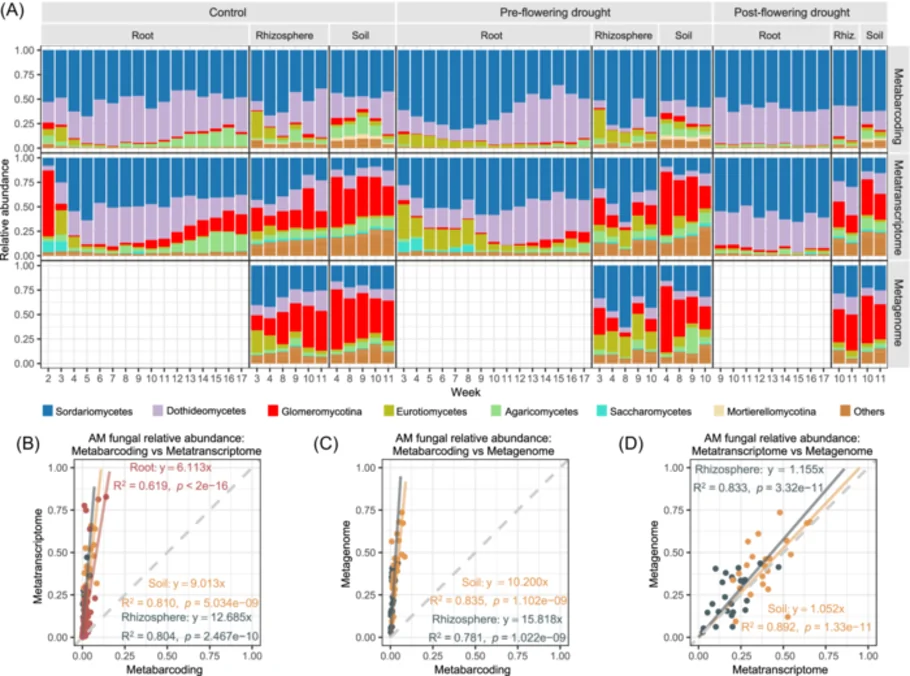
PCR‐free methods reveal higher Arbuscular mycorrhizal (AM) fungal relative abundance than metabarcoding. (A) AM fungal relative abundance as determined from metabarcoding, metatranscriptome, and metagenome datasets in root, rhizosphere, and soil in the control, the pre‐flowering drought, and the post‐flowering drought. Metatranscriptome and metagenome reads were translated into protein sequences and compared with databases, using the Kaiju method. (B) In PCR‐free metatranscriptomes, AM fungi exhibit higher relative abundance compared to metabarcoding in root (6:1), rhizosphere (12:1), and soil (9:1). (C) Similarly, PCR‐free metagenomes showed higher AM fungal relative abundance than metabarcoding in rhizosphere (15:1) and soil (9:1). (D) AM fungi were detected at comparable levels in PCR‐free metagenomes and metatranscriptomes.

Consistently across all compartments, AM fungal relative abundance was higher in metatranscriptomes than in ITS metabarcoding (ratios: 6:1 in roots, 12:1 in rhizosphere, and 9:1 in soil; Figure 1B–D). Similarly, metagenomes showed higher AM fungal relative abundance than metabarcoding in rhizosphere (15:1) and soil (9:1). Thus, AM fungi were underrepresented in ITS metabarcoding but detected at comparable levels in both PCR‐free methods (metagenome and metatranscriptome). Our root compartment findings in sorghum partially align with Chen et al., who reported lower Glomeromycota detection in ITS metabarcoding versus metatranscriptome in moss [19].

We propose that the underestimation of AM fungi in ITS metabarcoding likely stems from their lower rDNA copy numbers relative to other fungi. This interpretation is supported by Maeda et al. [31], who reported 11 rDNA copies for DAOM‐181602 (*Rhizoglomus irregulare*), whereas Lofgren et al. [32] noted typical copy numbers of ~80 for non‐AM fungi. However, fungal rDNA copy number data are very scarce, with information available for fewer than 100 fungal taxa and only one AM fungal species (DAOM‐181602). Thus, expanded rDNA copy number data, especially for AM fungi, are urgently needed to validate our hypothesis that rDNA copy number variation underlies the underestimation of AM fungi in metabarcoding relative to PCR‐free methods.

Despite differences in AM fungal detection between PCR‐free and PCR‐based methods, both metatranscriptome and metabarcoding data consistently indicated significant effects of drought and sorghum growth stage on root AM fungal relative abundance (Figure S5). As obligate symbionts, the dynamics of AM fungal relative abundance are likely regulated by the host plant. To investigate this relationship, we identified 284 sorghum genes positively correlated with AM fungal relative abundance (rho > 0.5, FDR *p* < 0.01; Figure 2A). Notably, 163 of these 284 genes overlapped with the 838 sorghum genes previously reported as upregulated upon AM fungal inoculation [33]. Furthermore, these 284 genes included key sorghum genes with validated functions essential for mycorrhizal symbiosis, such as those involved in fatty acid synthesis/transport (acyl‐ACP thioesterases (*Fatty Acid Transport Module*, FaTM), *Reduced Arbuscular Mycorrhiza* (RAM2), *Stunted Arbuscule* (STR)), strigolactone synthesis/transport (*DWARF 27* (D27), *Carotenoid Cleavage Dioxygenase 8* (CCD8)), and other key regulators (*Kinase 2* (KIN2), *Doesn't Make Infections 3* (DMI3), Cyclops, and *CYTOCHROME P450* (CYP722A1)) [34, 35, 36, 37, 38, 39, 40]. Given the established role of fatty acids in arbuscular mycorrhizal symbiosis [41] and our gene correlation results, we constructed a structural equation model (SEM) to depict interactions among drought, sorghum, and AM fungi. Our SEM indicated that AM fungal relative abundance was directly affected by sorghum development stage and indirectly affected by drought, mediated via sorghum fatty acid synthesis and transport pathways (involving FaTM, RAM2, and STR) (Figure 2B–E).

**Figure 2:**
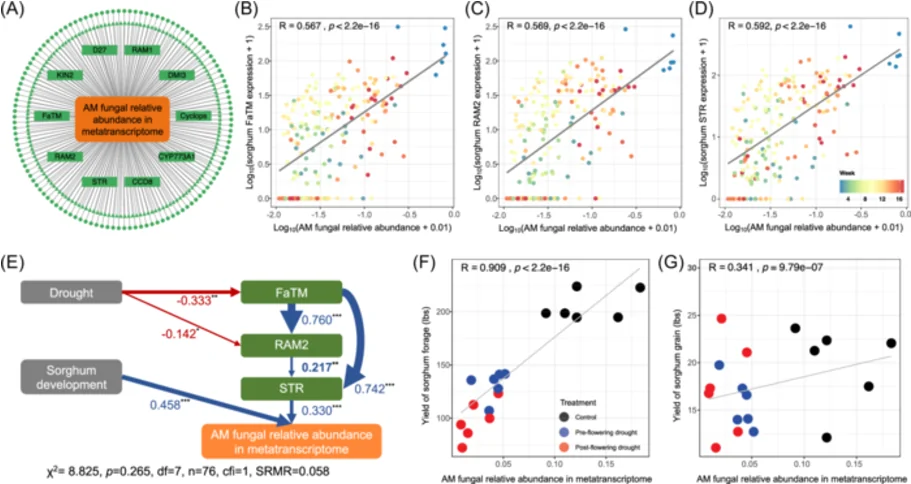
Arbuscular mycorrhizal (AM) fungal relative abundance in association with sorghum genes and productivity. (A) AM fungal relative abundance in relation to plant genes. Based on Spearman's correlation screening (rho > 0.5, FDR *p* < 0.01), we identified 284 plant genes (green nodes) significantly associated with AM fungal relative abundance (central orange rectangle node). Among these 284 genes, 163 genes (green triangles) overlapped with the 838 sorghum genes previously reported as upregulated upon AM fungal inoculation [33]. These 284 genes included key sorghum genes with validated functions essential for mycorrhizal symbiosis (green squares), such as those involved in fatty acid synthesis/transport (*Fatty Acid Transport Module* (FaTM), *Reduced Arbuscular Mycorrhiza* (RAM2), *Stunted Arbuscule* (STR)), strigolactone synthesis/transport (DWARF 27 (D27), *Carotenoid Cleavage Dioxygenase 8* (CCD8)), and other key regulators (*Kinase 2* (KIN2), *Doesn't Make Infections 3* (DMI3), CYCLOPS, and *Cytochrome P450* (CYP722A1)). (B−D) AM fungal relative abundance in relation to plant genes involved in fatty acid synthesis and transport: (B) FaTM, (C) RAM2, and (D) STR. (E) A structural equation model showing relationships among sorghum development stage, drought, AM fungal relative abundance, and plant genes involved in fatty acid synthesis and transport. AM fungal relative abundance is directly affected by sorghum development stage and indirectly affected by drought, mediated by sorghum fatty acid synthesis and transport pathways. Numbers above arrows indicate the magnitude of path coefficients (λ), depicted also by line width. Solid lines indicate significant pathways (*p < 0.05, **p < 0.01, ***p < 0.001). Model fit indices: cfi (comparative fit index), RMSEA (root mean square error of approximation), SRMR (standardized root mean square residual), df (degree of freedom). The gray background represents experimental design and sampling information, green represents plant genes, and orange represents AM fungal variables. Root AM fungal relative abundance detected by metatranscriptome shows significant correlations with both sorghum forage (F) and grain yield (G). The correlation between metabarcoding‐based AM fungal relative abundance and sorghum productivity indices is presented in Figures S6 and S7.

Is AM fungal relative abundance indicative of crop yield? We addressed this question by comparing root AM fungal relative abundance with forage yield, grain yield, weight‐of‐1000 sorghum seeds, and sorghum height that had been harvested at week 17 [42]. To provide a more accurate reflection of the role of AM fungi in the vigorous growth stage of sorghum, we calculated the average values of root AM fungal relative abundance from the 10th week to the 17th week. We found that sorghum forage yield was significantly positively correlated with root AM fungal relative abundance, whether estimated from metatranscriptomes (*R* = 0.91, *p* < 2.20 × e^−16^) or metabarcodes (*R* = 0.91, *p* < 2.20 × e^−16^) (Figure 2F,G and Figure S6). In addition, sorghum grain yield was significantly positively correlated with AM fungal relative abundance estimated from metatranscriptomes (*R* = 0.34, *p* = 9.79 × e^−7^) and metabarcodes (*R* = 0.37, *p* = 1.04 × e^−7^), as were the weight of 1000 seeds (metatranscriptomes: *R* = 0.41, *p* = 3.71 × e^−9^; metabarcodes: R = 0.58, *p* < 2.2 × e^−16^) and plant height (metatranscriptomes: *R* = 0.16, *p* = 0.03; metabarcodes: *R* = 0.30, *p* = 2.04 × e^−5^) (Figure 2F,G and Figures S6 and S7). Under drought, root AM fungal relative abundance correlated with the suppression of sorghum forage yield, grain yield, weight‐of‐1000 sorghum seeds, and sorghum height, with a stronger suppression caused by post‐flowering drought than by pre‐flowering drought (Figures 2F,G and Figures S6, S7). These findings suggest that root AM fungal relative abundance is a good indicator of sorghum forage yield, and can be a moderate indicator of sorghum grain yield and quality.

### AM fungal community gene expression profiles

We annotated AM fungal genes from 198 root RNA‐seq datasets by first removing sorghum and rRNA reads, then aligning the remaining reads to a non‐redundant catalog of 262,604 AM fungal genes derived from 18 high‐quality AM fungal genomes (Table S3). AM fungal read counts per sample ranged from 341 to 277,995 (median: 10,348; Figure S8). We found that AM fungal gene expression composition was significantly influenced by sampling time and drought treatment, but not by sorghum cultivar, and was significantly associated with AM fungal metabarcoding‐based taxonomic composition (Figures S9 and S10). The richness of transcribed AM fungal genes increased over time but decreased under drought, and showed positive correlations with both the richness and phylogenetic relatedness of AM fungal taxa (Figures S11 and S12). These patterns remained consistent across rarefaction depths of 1500 (94.44% samples retained), 3000 (81.82% retained), 5000 (70.71% retained), 8000 (58.08% retained), and 12,000 (43.43% retained) reads per sample (Figures S9 and S12).

During plant development, AM fungal genes involved in essential functions such as mating, sporulation, and resource acquisition increased in transcription (Figure S13). These functions correlated positively with AM fungal OTUs that gained in relative abundance over time and negatively with those that lost it (Figure S14). Besides, the richness of *Rhizoglomus*‐specific genes (identified by OrthoFinder) and *Rhizoglomus*‐specific core genes was higher in the later stage than in the early stage of plant development (Figure S15).

### Testing H_2_: The cytoskeleton organizations of plants and AM fungi are coordinated during plant development

To test H_2_, we identified AM fungal and sorghum genes whose expression was coupled, and from these genes, we then identified those involved in cytoskeleton function. Network analysis of gene expression in host plant roots found 99 AM fungal genes and 4712 plant genes that formed 14,812 significant positive and negative associations (*R* > 0.6 or *R* < −0.6, FDR *p* < 0.01) and supported four big modules (Figure 3A,B and Figure S16). The functional enrichment of the AM fungal genes that remained in the network or modules included: cytoskeleton, fatty acid metabolic process, meiotic and mitotic division, homologous chromosome segregation, cell polarity, oxidoreductase activity, organic acid metabolic process, and nucleoside binding (Figure 3C,D Figures S17 and S18). Note, expression of AM fungal genes involved in lipid metabolism correlated with expression of plant genes responsible for fatty acid synthesis and transport (Figure S19). For plant genes, enriched functions included: cytoskeleton, response to oxidative stress, transmembrane transport, cell cycle, DNA replication, protein phosphorylation, membrane, and response to stress (Figure 3C,D and Figures S17, S18). Previous research identified 838 sorghum genes upregulated when sorghum was inoculated with AM fungi in greenhouse experiments [33], and 267 of these genes were among the 1534 we found associated with AM fungal genes in network module #2 (Figure 3B). As aforementioned, among the 284 sorghum genes correlated with AM fungal relative abundance, 163 of them overlapped with the 838 sorghum genes upregulated when sorghum was inoculated with AM fungi in greenhouse experiments. Despite the differences in sampling time and frequency between our field study and the greenhouse study of Watts‐Williams et al. [35] (17 weekly samples vs. one sample at week 10), the large number of plant genes common to both studies indicate the relevance of our network analysis in detecting plant genes involved in AM symbiosis.

**Figure 3:**
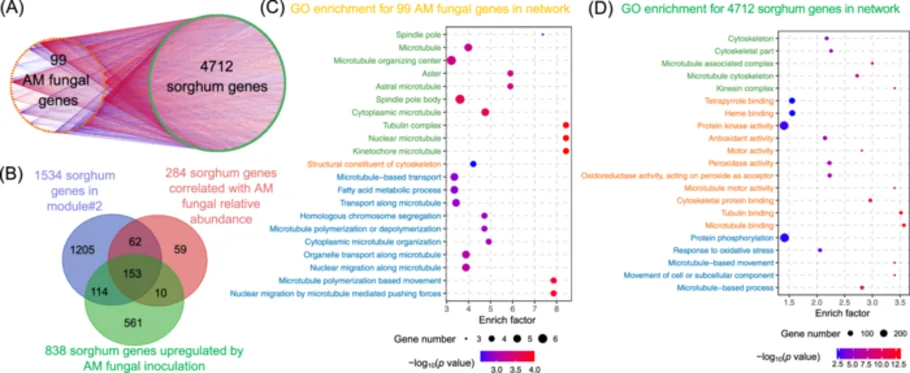
Plant and arbuscular mycorrhizal (AM) fungal genes responsible for cytoskeleton organization are enriched in the gene co‐expression network. (A) Network analysis revealed 14,812 significant co‐expressions involving 99 AM fungal genes and 4712 host plant root genes. This co‐expression network formed four major modules as visualized in Figure S16. (B) The shared and unique sorghum genes across the 1534 genes retained in module #2, the 284 genes correlated with AM fungal relative abundance, and the 838 genes previously shown to be upregulated after inoculation of sorghum with AM fungi. (C, D) Gene expression enrichment in the network shows the proportion of genes upregulated in a GO term (enrichment), the number of genes in the GO term, and the statistical significance of the enrichment judged by −log_10_(FDR), where high values are more significant. (C) The 99 AM fungal genes in the network showed enriched functions primarily associated with the cytoskeleton (GOs containing keywords: microtubule, tubulin complex, cytoskeleton). Beyond cytoskeleton, enriched AM fungal functions in network and modules also involved fatty acid metabolic process, meiotic and mitotic division, homologous chromosome segregation, cell polarity, oxidoreductase activity, organic acid metabolic process, and nucleoside binding. (D) The 4712 host plant genes in the network showed enriched functions primarily associated with the cytoskeleton (GOs containing keywords: microtubule, cytoskeleton, kinesin, tubulin). Beyond cytoskeleton, enriched plant functions in network and modules are also involved response to oxidative stress, transmembrane transport, cell cycle, DNA replication, protein phosphorylation, membrane, and response to stress. For the GO terms, blue typeface represents biological processes, orange represents molecular function, and green represents cellular component. Note that the functional enrichment of AM fungal and plant genes in each module is provided in Figures S17 and S18.

After limiting our scope to cytoskeleton‐related genes (based on GO terms for microtubule, tubulin, cytoskeleton, kinesin, and motor) that were enriched in both plant and AM fungal partners within the network, we focused our subsequent analysis on 102 plant and 98 AM fungal cytoskeletal genes (Figure 4A and Figures S17, S18). The heatmap of the expressions of plant and AM fungal genes related to cytoskeleton organization showed two distinct clusters (Figure 4A). Expression of the cluster of sorghum genes related to cytoskeleton organization was high in the early stage of plant development and decreased with time (Figure 4A). Expression of the cluster of AM fungal genes related to cytoskeleton organization was moderate in Week 2, low in Weeks 3–4, and high in the later stages (Weeks 6–17) (Figure 4A). Comparison of the expression of genes annotated to cytoskeleton organization of plant and AM fungi revealed a C‐shape relationship between them (Figure 4B). Specifically, the expression of plant and AM fungal cytoskeleton genes was positively correlated at Weeks 2–4, whereas the expression of plant and AM fungal cytoskeleton genes was not significantly correlated at Weeks 6–17 (Figure 4B and Figures S20, S21). Therefore, our H_2_ about the coordinated expression of plant and AM fungal genes of cytoskeleton organizations is supported at the early stage but not the later stage of plant development.

**Figure 4:**
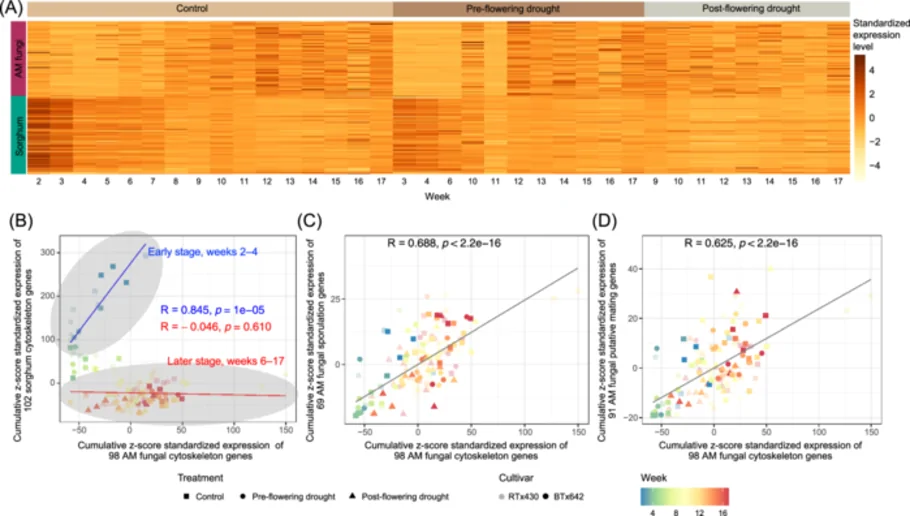
Cytoskeleton gene expression co‐occurrence in plant and arbuscular mycorrhizal (AM) fungal transcriptomes from roots over time. (A) The highest expression of cytoskeleton genes was found in early stages for plants and later stages for AM fungi. (B) Cumulative z‐score‐standardized expressions of plant and AM fungal cytoskeleton genes are strongly, positively correlated during the early stage of plant development but showed no significant correlation in later stages. Cumulative z‐score‐standardized expressions of AM fungal cytoskeleton genes strongly, positively correlated with those of (C) AM fungal clonal sporulation genes and (D) putative AM fungal sexual mating genes.

Could this co‐expression of cytoskeleton genes of AM fungi and their host plant merely reflect the plant life cycle and be independent from the succession of AM fungi? This scenario is unlikely, because a recent study showed that cytoskeleton‐associated processes were among the most strongly activated plant gene functions during the AM fungal accommodation process that takes place inside each colonized cell [43]. Thus, arbuscules provide the most likely explanation for the co‐expression of cytoskeleton genes of AM fungi and their host plant that we observe early in the development of arbuscular mycorrhiza. The involvement of the cytoskeletons of both partners has been demonstrated by a number of previous studies about the formation of arbuscules (Table S4) [23, 24, 26, 44]. And, as noted in the introduction, transcription studies using greenhouse plants have found positive associations between plant and AM fungal cytoskeleton genes, which have also been attributed to the formation of arbuscules [28, 29].

In later stages of development, we found a nonsignificant association between the expression of plant and AM fungal cytoskeleton genes that reflected an upregulation of AM fungal cytoskeleton genes without an upregulation of plant cytoskeleton genes (Figure 4A,B). A reduction in arbuscule formation as the annual plant ages can explain the lack of upregulation of cytoskeletal genes in the plant, but not the upregulation seen in the AM fungi. Upregulation of AM fungal cytoskeleton genes may correlate with the rounds of mitotic divisions needed to produce the many nuclei that populate the large, multinucleated, clonal spores produced by AM fungi [45, 46]. It is also possible that sexual mating is occurring at this time, as both types of reproduction depend strongly on the cytoskeleton.

To further investigate AM fungal reproduction, we focused on the expression of AM fungal genes involving the fungal mating response and sporulation. Aware of the controversy over the identity of AM fungal genes orthologous to validated fungal mating genes [47, 48], we curated a list of AM fungal genes using the fungal‐mating‐related, hidden Markov model (HMM) in the Pfam database (Table S5), which we augmented with genes involved in sporulation. Our results showed that the expression of genes involved both in sporulation and sexual reproduction increased with time (Figures S13 and S22). Not surprisingly, we then found that expression of AM fungal genes involved with the cytoskeleton was strongly positively correlated with expression of AM fungal genes involved with sporulation and mating (Figure 4C,D, and Figures S23, S24), and these patterns were robust across the three drought treatments and two sorghum cultivars. Note that the function of sexual mating was enriched relative to other genes both in the network as a whole (homologous chromosome segregation; Figure 3C,D) as well as in the network module #2 (meiotic cell cycle; Figure S18). This enrichment indicates that expression of mating genes is more than simply the byproduct of the massive upregulation of the AM fungal gene repertoire (Figure S12).

## 3 CONCLUSION

In summary, although ITS metabarcoding has been the predominant method for characterizing fungal communities [49, 50, 51, 52, 53], our field‐based multi‐omics analysis strongly suggests that AM fungal relative abundances have been systematically underestimated in previous studies. This methodological advancement revealed a surprisingly strong correlation between AM fungal relative abundance and sorghum productivity, pointing toward the potential to artificially modulate AM fungal communities to enhance crop growth and yield. Furthermore, integrated co‐expression network analysis uncovered that symbiotic life history from arbuscule formation to sporulation is governed by coupled and decoupled co‐expression of plant and AM fungal genes.

## 4 METHODS

### Experimental design

This research combines and re‐analyzes previously published multi‐omics datasets collected in semiarid agricultural fields. The details on experimental design, sampling, and sequencing can be found in those studies [15, 42, 54, 55, 56]. Briefly, we collected samples of roots, rhizosphere and soil each week over the 17 week sorghum growing season of a drought manipulation experiment including three treatments: (1) control, regularly watered every week; (2) pre‐flowering drought, not watered until the ninth week and then watered regularly; and (3) post‐flowering drought, regular irrigation until the 10th week and then ceasing watering [42]. DNA‐based ITS2 metabarcoding sequencing [15] was performed on root, rhizosphere, and soil samples (Figure S1). Poly(A)‐based RNA‐seq was performed on 198 root samples (Figure S1). Additionally, shotgun metagenome and metatranscriptome sequencing were performed on selected rhizosphere and soil samples (Figure S1). To quantify the fungal and AM fungal reads, the Kaiju tool [57] was respectively applied to the root RNA‐seq, rhizosphere and soil metagenome, and rhizosphere and soil metatranscriptome datasets, by translating nucleic acid sequences into amino acid sequences and comparing them against protein databases for taxonomic assignment.

### AM fungal community profiling via ITS2 metabarcoding

Fungal OTUs [58] belonging to Glomeromycotina were identified as arbuscular mycorrhizal fungi (Table S2) [15]. Details on ITS2 metabarcoding‐based characterization of AM fungal communities have been previously published [10, 54]. Briefly, DNAs that had been extracted using the MoBio PowerSoil DNA kit (MoBio) were used as templates for ITS2 amplification using dual‐barcoded 5.8SFun and ITS4Fun primers. The 5.8SFun and ITS4Fun primer pair was designed to match Glomeromycotina lineages based on alignments of published SSU‐ITS‐LSU sequences [59]. The utility of this primer pair has been demonstrated by its detection of numerous Glomeromycotina, including species of *Acaulosporaceae*, *Archaeosporaceae*, *Diversisporaceae*, *Gigasporaceae*, *Glomeraceae*, and *Paraglomeraceae* [60, 61, 62, 63]. Additionally, the 5.8SFun and ITS4Fun primers also aligned well with the newly introduced AM fungal lineages as described in Tedersoo et al. [30] (Figure S25). Equal amounts of PCR product from each sample were pooled, purified, and sequenced on the Illumina MiSeq PE300 sequencing platform at the Vincent J. Coates Genomics Sequencing Laboratory (GSL, University of California) [54]. The raw sequences were analyzed using the USEARCH v8.0 pipeline [64] embracing steps of quality control, primer and adapter removal, merging, desingleton, and clustering processes. The OTUs were identified by blastn referencing the EUKARYOME database v1.8 [65]. The OTU table was rarefied to 68 reads per sample, and AM fungal OTU richness index was calculated by summing the number of OTUs in a specific sample. To elucidate the phylogenetic placements of the AM fungal OTUs, representative sequences of these OTUs were aligned with identified AM fungal SSU‐ITS‐LSU sequences obtained from the EUKARYOME database v1.8 [65], and the combined dataset was used to construct a phylogenetic tree using Galaxy v22.05 (https://galaxy.pasteur.fr/) for multiple alignment using fast Fourier transform (MAFFT) alignment, block mapping and gathering with entropy cleaning, and MrBayes tree generation [30, 66]. AM fungal indicator species for selected sampling times were identified, and their threshold values were calculated using threshold indicator species analyses (TITAN) in the TITAN2 package v2.4.3. To evaluate the phylogenetic relatedness of AM fungal OTUs within each sample, the net relatedness index was computed using the AM fungal phylogenetic trees and community composition data, utilizing the “ses.mpd” (× −1) command in the picante package v1.8.2 [67].

### Profiling the functional dynamics of AM fungal communities using metatranscriptomics

The raw sorghum root RNA‐seq sequences underwent quality control using fastp v0.12.4 [68]. High‐quality reads were then processed to detect and filter out sorghum genes using bowtie2 v2.5.3 [69], aligned against the *Sorghum bicolor* v3.1.1 transcript reference from Phytozome v13 [70]. Remaining reads were further filtered to remove rRNA‐related sequences using ribodetector v0.2.7 [71]. These steps produced purified read pools with a median of 2,869,156 reads per sample (range: 1,108,417−9,235,720). The mRNAs retained in the root RNA‐seq dataset were annotated against a non‐redundant reference gene catalog (262,604 genes) derived from 15 high‐quality AM fungal transcriptomic datasets sourced from MycoCosm (https://mycocosm.jgi.doe.gov/mycocosm/home) and 3 additional datasets from the NCBI platform (Table S3). Annotation was also performed by referencing a *Rhizoglomus*‐specific gene catalog (comprising genes occurring exclusively in *Rhizoglomus* spp. genomes as detected by OrthoFinder v3.0.1b1 [72]), and a *Rhizoglomus*‐specific core gene catalog (comprising genes occurring exclusively in more than 75% of *Rhizoglomus* spp. with available genomes).

This process identified AM fungal reads ranging from 341 to 277,995 among the 198 samples (median: 10,348; Figure S8). To mitigate sequencing depth variability, rarefaction‐based normalization was performed by subsampling mapped reads to standardized depths of 1500 (94.44% samples retained), 3000 (81.82% retained), 5000 (70.71% retained), 8000 (58.08% retained), and 12,000 (43.43% retained) reads per sample. All subsampling thresholds yielded consistent trends in relationships between AM fungal gene expression richness, taxonomic diversity, and phylogenetic relatedness (Figure S12). Subsequent analyses focused on the 5000‐read subsampled dataset to balance sample retention (70.71% retained) and functional gene detection. The expression levels of AM fungal genes were estimated using the Salmon v1.10.1 method [73], in which the transcript abundance was calculated as transcripts per million values to account for technical variations in gene length and residual sequencing depth effects.

Functions of AM fungal genes were identified by aligning the transcripts to the EggNOG v5.0.2 database [74] using the Diamond v2.1.8.162 method [75]. In addition, functions of candidate genes were annotated by referencing Pfam using the HMMER v3.3.2 method [76], with a focus on the transporters of amino acid, ammonium, fatty acid, phosphorus, and sugar, as well as aquaporins and genes involved in sporulation and mating. Mindful of the controversy over AM fungal putative mating genes, we compiled a collection of putative AM fungal mating genes using aforementioned Pfam‐method, namely that published by Mateus et al. [77], and that provided by Mathu Malar and Nicolas Corradi [78]. To generate a standardized index for cross‐comparison of functional profiles in AM fungal or sorghum systems, we normalized individual gene expression values using z‐score transformation (mean subtraction followed by division by the standard deviation). These normalized scores, which are relative and can have negative values, were aggregated to compute composite functional indices for cytoskeleton and AM fungal‐associated processes, including amino acid transporters, ammonium transporter, aquaporin, fatty acid transporters, sporulation‐specific genes, and mating type genes.

### Constructing the structural equation model

For both PCR‐free and ITS metabarcoding sequences, the percentages of AM fungal reads out of all fungal reads were calculated and subjected to linear regression. Spearman's correlation was performed to detect plant genes related to root AM fungal relative abundance. To depict the complex interactions among drought, plant, and AM fungi, a SEM was constructed using the lavaan v0.6‐15 [79] package. Based on a priori and theoretical knowledge, we assumed a conceptual model, in which drought and sorghum development affect plant genes responsible for fatty acid synthesis and transport. These fatty acid genes, in turn, influence AM fungal relative abundance. A maximum likelihood estimation method was used to compare the SEM models with observations. Model adequacy was determined by Chi‐square tests, comparative fit index, root mean square error of approximation, standardized root mean square residual, and degree of freedom (df) in the lavaan v0.6‐15 package [79].

We employed Pearson's correlation to investigate the link of root AM fungal relative abundance with sorghum forage yield, grain yield, weight‐of‐1000 sorghum seeds, and sorghum height that had been harvested at week 17 [42]. To provide a more accurate reflection of the role of AM fungi in the vigorous growth stage of sorghum, we calculated the average values of root AM fungal relative abundance from the 10th week to the 17th week.

Principal coordinate (PCo) analysis followed by permutational analysis of variance (permANOVA) was performed to test and visualize the variation in AM fungal transcriptome composition as depicted by Bray–Curtis dissimilarity in the Ape v5.7‐1 [80] and vegan v2.6‐4 packages. The correlation between AM fungal transcriptome and community compositions was investigated by applying the Procrustes test to PCo vectors and applying the Mantel test to Bray–Curtis dissimilarity values using the ecodist v2.0.9 [81] and vegan v2.6‐4 packages. AM fungal gene expression richness was calculated using the specnum command in vegan v2.6‐4.

### Constructing a co‐expression network

To examine the potential interaction between plants and AM fungi, a co‐expression network of AM fungal and sorghum genes was constructed based on Spearman's correlation (rho > 0.6 or rho < −0.6; FDR *p* < 0.01) using the psych v2.3.3 and igraph v2.0.3 package [82]. Network modules were detected and visualized using the cluster_fast_greedy command in the igraph v2.0.3 package. To depict gene functions, functional enrichment was performed for both AM fungal and sorghum genes in the network and in each module using the topGO v2.50.0 package and agriGO v2 (https://systemsbiology.cau.edu.cn/agriGOv2/) [83].

Having found that the cytoskeleton‐related function was enriched in different modules for AM fungal genes and plant genes, a heatmap was constructed to depict the expressions of plant and AM fungal cytoskeleton genes over time and treatment using the pheatmap v1.0.12 package. The relationship between the expressions of AM fungal and plant cytoskeleton genes was visualized using a scatterplot in ggplot2 v3.4.1 package [84]. To infer the function of cytoskeleton gene expression upregulation, the gene expression of cytoskeleton organization genes was regressed against AM fungal sporulation genes expression, and AM fungal putative mating gene expression. All statistical analyses were performed in R v4.2.3, except for functional enrichment using agriGO v2 (https://systemsbiology.cau.edu.cn/agriGOv2/).

## AUTHOR CONTRIBUTIONS

**Peilin Chen:** Conceptualization; methodology; software; writing—review and editing; writing—original draft; validation. **John W. Taylor:** Conceptualization; project administration; supervision; investigation; writing—review and editing; resources. **Cheng Gao:** Conceptualization; investigation; funding acquisition; writing—review and editing; project administration; supervision; resources. All authors have read the final manuscript and approved it for publication.

## CONFLICT OF INTEREST STATEMENT

The authors declare no conflicts of interest.

## ETHICS STATEMENT

No animals or humans were involved in this study.

## Supporting information


**Figure S1:** Omics datasets.
**Figure S2:** The phylogeny of ITS2 sequences of arbuscular mycorrhizal fungal operational taxonomical units (OTUs).
**Figure S3:** Temporal dynamics of arbuscular mycorrhizal (AM) fungal operational taxonomic units (OTUs), community composition, richness, and phylogenetic relatedness.
**Figure S4:** Relative abundances of fungal and arbuscular mycorrhizal (AM) fungal gene expressions in roots in terms of time and drought.
**Figure S5:** Arbuscular mycorrhizal (AM) fungal relative abundance in relation to time and drought treatment.
**Figure S6:** The correlation between metabarcoding‐based arbuscular mycorrhizal fungal relative abundance and sorghum productivity.
**Figure S7:** The correlation between metatranscriptome‐based root arbuscular mycorrhizal fungal relative abundance and sorghum productivity.
**Figure S8:** Distribution of the number of arbuscular mycorrhizal fungal reads across 198 root RNA‐seq samples.
**Figure S9:** The associations of arbuscular mycorrhizal (AM) fungal metatranscriptomic composition with time, drought, and host plant and that with AM fungal community composition.
**Figure S10:** Krona charts showing the relative abundance of root arbuscular mycorrhizal (AM) fungal taxa based on RNA‐seq transcripts annotated with Kaiju and ITS2 DNA metabarcoding.
**Figure S11:** Temporal changes in relative abundances of KEGG Orthologs (KOs) in arbuscular mycorrhizal fungal metatranscriptome along with plant development.
**Figure S12:** The richness of arbuscular mycorrhizal (AM) fungal gene expressed is strongly associated with AM fungal OTU richness and the phylogenetic net relatedness index.
**Figure S13:** Upregulation of arbuscular mycorrhizal fungal genes with key functions as the plant matures over time.
**Figure S14:** Heatmap showing correlations between arbuscular mycorrhizal (AM) fungal operational taxonomic units (OTUs) and AM fungal transcriptomic measures.
**Figure S15:** The richness of *Rhizoglomus*‐specific genes expression.
**Figure S16:** A co‐expression network of arbuscular mycorrhizal fungal and sorghum genes was constructed based on Spearman's correlation.
**Figure S17:** Functional enrichment of the plant and arbuscular mycorrhizal (AM) fungal genes retained in the module #1 of the co‐expression network.
**Figure S18:** Functional enrichment of the plant and arbuscular mycorrhizal (AM) fungal genes retained in the module #2 of the co‐expression network.
**Figure S19:** The expressions of arbuscular mycorrhizal fungal lipid metabolic genes correlates positively with expression of plant genes involved in synthesis and transporters of fatty acids.
**Figure S20:** Heatmap displaying the massive positive pairwise correlations between 98 arbuscular mycorrhizal (AM) fungal cytoskeleton genes and 102 sorghum cytoskeleton genes during the early developmental stage (weeks 2–4).
**Figure S21:** The heatmap of pairwise correlations revealed that both significant positive (351) and negative (410) correlations were detected between 98 arbuscular mycorrhizal (AM) fungal cytoskeleton genes and 102 sorghum cytoskeleton genes during later developmental stages (weeks 6–17).
**Figure S22:** The upregulation of arbuscular mycorrhizal fungal putative mating genes and clonal sporulation genes with time.
**Figure S23:** Heatmap displaying the massive positive pairwise correlations between 98 arbuscular mycorrhizal (AM) fungal cytoskeleton genes and 69 AM fungal sporulation genes.
**Figure S24:** Heatmap displaying the massive positive pairwise correlations between 98 arbuscular mycorrhizal (AM) fungal cytoskeleton genes and 91 AM fungal putative mating genes.
**Figure S25:** The alignments of the 5.8SFun and ITS4Fun primers with the newly introduced arbuscular mycorrhizal fungal groups.

**Table S1:** A list of studies investigating fungal genes from plant transcriptomes.
**Table S2:** The taxonomy of arbuscular mycorrhizal fungal operational taxonomical units.
**Table S3:** Arbuscular mycorrhizal fungal genomes used to annotate metatranscriptome dataset.
**Table S4:** A list of earlier studies finding changes of plant functions involving cytoskeleton reorganization in response to arbuscular mycorrhizal fungal inoculation.
**Table S5:** A compiled list of AM fungal putative mating genes.

## Data Availability

No new data were generated in this study. All data have been previously deposited in NCBI (i) GenBank (representative AM fungal read set) with accession codes MG008508‐MG008559, (ii) Sequence Read Archive (fungal raw data) with accession codes Bioproject PRJNA412410 (https://www.ncbi.nlm.nih.gov/bioproject/PRJNA412410/) and PRJNA494573 (https://www.ncbi.nlm.nih.gov/bioproject/PRJNA494573/), (iii) Gene Expression Omnibus database (GEO) with accession code GSE128441 (https://www.ncbi.nlm.nih.gov/geo/query/acc.cgi?acc=GSE128441), and (iv) Metagenome and metatranscriptome data with accession codes Bioproject PRJNA435634 (https://www.ncbi.nlm.nih.gov/bioproject/PRJNA435634/) and PRJNA435642 (https://www.ncbi.nlm.nih.gov/bioproject/PRJNA435642/). The data and source code implementing the analyses in this manuscript will be available on Github https://github.com/chenpl2023/Sorghum-Roots-AMF-transcript. Supplementary materials (figures, tables, graphical abstract, slides, videos, Chinese translated version, and update materials) may be found in the online DOI or iMetaOmics Science http://www.imeta.science/imetaomics.
