# Supplementary material for: Arbuscular mycorrhizal fungal community abundance, functions, and symbiotic interactions revealed by root metatranscriptomes
Source: IMetaOmics. 2026 Feb 21;3(2):e70087. doi: 10.1002/imo2.70087 (PMC13674444; doi:10.1002/imo2.70087)
Supplement: Supplementary file 1 [file IMO2-3-e70087-s002.docx]

Supporting Information to

**Arbuscular mycorrhizal fungal community abundance, functions, and symbiotic interactions revealed by root metatranscriptomes**

**Running title**: AM fungal symbiosis decoded by root metatranscriptomes

Peilin Chen^1,2^, John W. Taylor^3^, Cheng Gao^1,2*^

^1^State Key Laboratory of Microbial Diversity and Innovative Utilization, Institute of Microbiology, Chinese Academy of Sciences, Beijing 100101, China

^2^College of Life Sciences, University of Chinese Academy of Sciences, Beijing 100049, China

^3^Department of Plant and Microbial Biology, University of California, Berkeley, CA 94720, USA

*Correspondence: [gaoc@im.ac.cn](mailto:gaoc@im.ac.cn) (Cheng Gao)

**Figure S1** **Omics datasets.** Root, rhizosphere, and soil samples were collected weekly over a growing season from a semiarid agricultural field under control, pre-flowering drought, or post-flowering drought treatments. This study includes sorghum RNA-seq and internal transcribed spacer 2 (ITS2) metabarcoding data from root samples collected from time points TP02−TP17, and shotgun metagenome, metatranscriptome and ITS2 metabarcoding data from rhizosphere and soil samples collected in time points TP04, TP08, TP10, and TP11.

**Figure S2** **The phylogeny of ITS2 sequences of 65 arbuscular mycorrhizal (AM) fungal operational taxonomical units (OTUs) obtained in this study, combined with named sequences from EUKARYOME database v1.8 (Tedersoo *et al*. [1]).** The AM fungal phylogenetic tree and DNA sequence alignment were deposited in TreeBASE (study accession URL: http://purl.org/phylo/treebase/phylows/study/TB2:S31873?x-access-code=e860bc538ca712ba46fbbe7591308c68&format=html).

**Figure S3** **Temporal dynamics of arbuscular mycorrhizal (AM) fungal operational taxonomic units (OTUs), community composition, richness, and phylogenetic relatedness.** (A) The phylogeny of 19 AM fungal OTUs exhibiting significant temporal changes in relative abundances. Note that this phylogeny is a subset of the phylogeny of 65 AM fungal OTUs as detailed in Figure S2. (B) Significant temporal changes in relative abundances of 19 AM fungal OTUs as detected by threshold indicator taxa analyses (TITAN). For each OTU, the genus name is noted by color and filled symbols show declining abundance (z−), and open symbols show increasing abundance (z+). (C) The mean relative abundance of these 19 AM fungal OTUs exhibiting significant temporal changes. (D) AM fungal OTUs richness increased with time. (E) AM fungal phylogenetic relatedness (net relatedness index, NRI) increases over time. (F) AM fungal community composition is influenced by, in order of importance, time, treatment, and host cultivar, based on principal coordinate (PCo) analysis with permutational analysis of variance (PERMANOVA). Vectors in the ordination plot depict the 15 AM fungal OTUs significantly correlated with the first two PCo axes and these OTUs represent a subset of the 19 AM fungal OTUs exhibiting significant temporal changes as detected by TITAN. The PCo analysis of AM fungal community composition (DNA-metabarcoding), together with the PCo analysis of AM fungal functional composition (RNA-seq) of the same samples (Figure S9), was then subjected to Procrustes testing to investigate the correlation between these two datasets (Figure S9). Note that the PCo analysis of AM fungal community composition presented here used a subset of a larger number of samples previously published in Gao *et al*. [2,3]. (G) The relationships between time and the relative abundances of 19 AM fungal OTUs. Different colors represent different genera. The three treatments are represented by shapes, with squares for control, circles for pre-flowering drought, and triangles for post-flowering drought. The two sorghum cultivars are represented by different levels of transparency, with light color for RTx430 and dark color for BTx642.

**Figure S4** **Relative abundances of fungal and arbuscular mycorrhizal (AM) fungal gene expressions in roots in terms of time and drought.** (A) The count of AM fungal reads is the number of AM fungal reads identified from the RNA-seq data after being translated into protein sequences and comparison with databases using the Kaiju method. (B) Fungal relative abundance is the percent of fungal reads identified from the RNA-seq data. (C) AM fungal relative abundance is the percent of AM fungal reads identified from the RNA-seq data. (D) The ratio of AM fungal reads to fungal reads is the percent of AM fungal reads out of all fungal reads identified from the root RNA-seq data.

**Figure S5 Arbuscular mycorrhizal (AM) fungal relative abundance in relation to time and drought treatment.** (A) Metatranscriptome-based AM fungal relative abundance was significantly affected by time, drought treatment, and sorghum cultivar. (B) Metabarcoding-based AM fungal relative abundance was significantly affected by time and drought treatment, but not by sorghum cultivar.

**Figure S6 The correlation between metabarcoding-based** **arbuscular mycorrhizal fungal relative abundance and sorghum productivity.** (A) forage yield, (B) grain yield, (C) 1000-seed weight, and (D) plant height in sorghum.

**Figure S7 The correlation between metatranscriptome-based root arbuscular mycorrhizal fungal relative abundance and sorghum productivity.** (A) 1000-seed weight and (B) plant height in sorghum.

**Figure S8 Distribution of the number of arbuscular mycorrhizal fungal reads across 198 root RNA-seq samples.** To mitigate sequencing depth variability, rarefaction-based normalization was performed by subsampling mapped reads to standardized depths of 1500 (94.44% samples retained), 3000 (81.82% retained), 5000 (70.71% retained), 8000 (58.08% retained), and 12,000 (43.43% retained) reads per sample. The main figures presented results of based on the subsampling threshold of 5000 reads, and the results based on other subsampling thresholds are presented in Figures S9 and S12.

**Figure S9 The associations of** **arbuscular mycorrhizal (AM) fungal metatranscriptomic composition with time, drought, and host plant (A–E), and that with AM fungal community composition (F–J)** across subsampled datasets with the thresholds of 1500 (A and F), 3000 (B and G), 5000 (C and H), 8000 (D and I), and 12,000 (E and J).

**Figure S10 Krona charts showing the relative abundance of root arbuscular mycorrhizal (AM) fungal taxa based on (A) RNA-seq transcripts annotated with Kaiju and (B) ITS2 DNA metabarcoding.** Note in addition to the latest version of AM fungal taxonomy [1] for metabarcoding-derived taxa, parenthetical notations show classifications comparable to Kaiju annotations. Interactive versions are available at GitHub (https://github.com/chenpl2023/Sorghum-Roots-AMF-transcript).

**Figure S11 Temporal changes in relative abundances of KEGG Orthologs** **(KOs) in arbuscular mycorrhizal fungal metatranscriptome** **along with plant development.**

**Figure S12** **The richness of arbuscular mycorrhizal (AM) fungal gene expressed is strongly associated with AM fungal OTU richness and the phylogenetic net relatedness index.** The richness of expressed AM fungal genes increased over time (A–E) and correlated with both AM fungal operational taxonomic unit (OTU) richness (F–J) and net relatedness index (NRI) (K–O). These patterns were detected in subsamples using AM fungal read thresholds of 1500 (A, F, K), 3000 (B, G, I), 5000 (C, H, M), 8000 (D, I, N), and 12,000 (E, J, O).

**Figure S13** **Upregulation of arbuscular mycorrhizal fungal genes with key functions as the plant matures over time.** Functions of candidate genes were annotated by referencing Pfam using the HMMER method. AAT, amino acid transporter; AMT, ammonium transporter; AQPF, aquaporin protein family; FAT, fatty acid transporter; MAT, mating type genes; PT, phosphate transporter; SPS, sporulation-specific genes; ST, sugar transporter.

**Figure S14** **Heatmap showing correlations between arbuscular mycorrhizal (AM) fungal operational taxonomic units (OTUs) and AM fungal transcriptomic measures.** The left panel is the phylogeny of 17 AM fungal OTUs exhibiting significant (*p* < 0.01) correlation with levels of AM fungal transcriptomic measures, including AM-fungi/all-fungi in RNA-seq, AM fungal gene richness, and cumulative z-score-standardized expressions of amino acid transporter (AAT), ammonium transporter (AMT), aquaporin (AQPF), fatty acid transporter (FAT), mating type genes (MAT), phosphate transporter (PT), sporulation-specific genes (SPS), and sugar transporter (ST). Pearson’s correlations with an FDR-adjusted *p* value of less than 0.01 are depicted by the color depth (blue for positive correlations and red for negative correlations), square size, and correlation coefficient. Note that this phylogeny is a subset of the phylogeny of 65 AM fungal OTUs as detailed in Figure S2.

**Figure S15** **The richness of *Rhizoglomus*-specific genes expression.** (A) The richness of *Rhizoglomus*-specific genes expression was higher in the later stage than in the early stage of plant development. (B) The richness of *Rhizoglomus*-specific core genes (those occurring in more than 75% of *Rhizoglomus* spp. with available genomes) was also higher in the later stage than in the early stage.

**Figure S16** **A co-expression network of arbuscular mycorrhizal fungal and sorghum genes was constructed based on Spearman’s correlation (rho > 0.6 or rho < −0.6; FDR *p* < 0.01).** Network modules were detected and visualized using the cluster_fast_greedy command in the igraph package v2.0.3. Different colors identify the different gene network modules.

**Figure S17** **Functional enrichment of the plant and arbuscular mycorrhizal** **(AM) fungal genes retained in the module #1 of the co-expression network.** (A) AM fungal genes (*n* = 25) in the network module #1 showing enriched functions primarily associated with oxidoreductase, lipid, fatty acid, organic acid metabolic processes. (B) Host plant genes (*n* = 2337) in the network module #1 showing enriched functions primarily associated with cytoskeleton organization. For the GO terms, blue typeface represents biological processes, orange represents molecular function, and green represents cellular component. This figure on network module#1 is supplementary to Figure 3 that presented the co-expression network as a whole.

**Figure S18** **Functional enrichment of the plant and arbuscular mycorrhizal (AM) fungal genes retained in the module #2 of the co-expression network.** (A) AM fungal genes (*n* = 64) in the network module #2 showing enriched functions primarily associated with cytoskeleton organization. (B) Host plant genes (*n* = 1534) in the network module #2 showing enriched functions primarily associated with transmembrane transport, response to wounding, proteolysis, and kinase. For the GO terms, blue typeface represents biological processes, orange represents molecular function, and green represents cellular component. This figure on network module#2 is supplementary to Figure 3 that presented the co-expression network as a whole.

**Figure S19 Cumulative z-score-standardized** **expressions of arbuscular mycorrhizal fungal lipid metabolic genes correlates positively with expression of plant genes involved in synthesis and transporters of fatty acids**, including *Fatty Acid Transport Module*, (FaTM), *Reduced Arbuscular Mycorrhiza 2* (RAM2), *Stunted Arbuscle* (STR), and *Stunted Arbuscle* 2b (STR2b).

**Figure S20** **Heatmap displaying the massive positive pairwise correlations between 98 arbuscular mycorrhizal (AM) fungal cytoskeleton genes and 102 sorghum cytoskeleton genes** **during the early developmental stage (weeks 2–4).** Color intensity represents the strength of the Pearson’s correlation, with blue indicating positive correlations and red indicating negative correlations. Only statistically significant correlations (FDR-adjusted *p*-value < 0.01) are shown. This figure is supplementary to Figure 4B.

**Figure S21** **The heatmap of pairwise correlations revealed that both significant positive (351) and negative (410) correlations were detected between 98 arbuscular mycorrhizal (AM) fungal cytoskeleton genes and 102 sorghum cytoskeleton genes during later developmental stages (weeks 6–17).** Color intensity corresponds to the strength of the Pearson’s correlation, with blue denoting positive correlations and red representing negative correlations. Only statistically significant correlations (FDR-adjusted *p*-value < 0.01) are displayed. This figure is supplementary to Figure 4B.

**Figure S22** **The upregulation of arbuscular mycorrhizal fungal (A) putative mating genes and (B) clonal sporulation genes with time.**

**Figure S23** **Heatmap displaying the massive positive pairwise correlations between 98 arbuscular mycorrhizal (AM) fungal cytoskeleton genes and 69** **AM fungal sporulation genes.** Color intensity represents the strength of the Pearson’s correlation, with blue indicating positive correlations and red indicating negative correlations. Only statistically significant correlations (FDR-adjusted *p*-value < 0.01) are shown. This figure is supplementary to Figure 4C.

**Figure S24** **Heatmap displaying the massive positive pairwise correlations between 98 arbuscular mycorrhizal (AM) fungal cytoskeleton genes and 91 AM fungal** **putative mating genes.** Color intensity represents the strength of the Pearson’s correlation, with blue indicating positive correlations and red indicating negative correlations. Only statistically significant correlations (FDR-adjusted *p*-value < 0.01) are shown. This figure is supplementary to Figure 4D.

**Figure S25** **The alignments of the 5.8SFun and ITS4Fun primers with the newly introduced arbuscular mycorrhizal fungal groups in Tedersoo *et al.* [4].** Note that we failed to find the LSU-ITS-SSU sequences of *Kahvenaceae*, *Lehetuaceae*, *Nikkaluoktaceae*, *Parniguaceae*, *Riederbergaceae*, *Tammsaareaceae*, *Unemaeeaceae*, and *Vinositunicaceae* from EUKARYOME database v1.8 [1].

**REFERENCES**

1. Tedersoo, Leho, Mahdieh S. Hosseyni Moghaddam, Vladimir Mikryukov, Ali Hakimzadeh, Mohammad Bahram, R. Henrik Nilsson, Iryna Yatsiuk, et al. 2024. “EUKARYOME: the rRNA gene reference database for identification of all eukaryotes.” *Database* 2024: baae043. https://doi.org/10.1093/database/baae043

2. Gao, Cheng, Liliam Montoya, Ling Xu, Mary Madera, Joy Hollingsworth, Elizabeth Purdom, Robert B. Hutmacher, et al. 2019. “Strong succession in arbuscular mycorrhizal fungal communities.” *The ISME Journal* 13: 214-226. https://doi.org/10.1038/s41396-018-0264-0

3. Gao, Cheng, Pierre-Emmanuel Courty, Nelle Varoquaux, Benjamin Cole, Liliam Montoya, Ling Xu, Elizabeth Purdom, et al. 2023. “Successional adaptive strategies revealed by correlating arbuscular mycorrhizal fungal abundance with host plant gene expression.” *Molecular Ecology* 32: 2674-2687. https://doi.org/10.1111/mec.16343

4. Tedersoo, Leho, Franco Magurno, Saad Alkahtani, Vladimir Mikryukov. 2024. “Phylogenetic classification of arbuscular mycorrhizal fungi: new species and higher-ranking taxa in *Glomeromycota* and Mucoromycota (class Endogonomycetes).” *MycoKeys* 107: 273-325. https://doi.org/10.3897/mycokeys.107.125549
